# Role of chemokines CXCL9, CXCL10, CXCL11, and CXCR3 in the serum and minor salivary gland tissues of patients with Sjögren’s syndrome

**DOI:** 10.1007/s10238-024-01401-4

**Published:** 2024-06-20

**Authors:** Ji-Won Kim, Mi-Hyun Ahn, Ju-Yang Jung, Chang-Hee Suh, Jae Ho Han, Hyoun-Ah Kim

**Affiliations:** 1 Department of Rheumatology, Ajou University School of Medicine, 164 Worldcup-ro, Yeongtong-gu, Suwon, 16499 Republic of Korea; 2 Department of Pathology, Ajou University School of Medicine, 164 Worldcup-ro, Yeongtong-gu, Suwon, 16499 Republic of Korea

**Keywords:** Sjögren’s syndrome, Chemokine, Biomarker

## Abstract

This study aimed to investigate the serum and expression levels of C-X-C motif chemokine ligand 9 (CXCL9), CXCL10, CXCL11, and CXC receptor 3 (CXCR3) in minor salivary glands (MSGs) of patients with primary Sjögren’s syndrome (pSS), and to explore their correlations with clinical parameters. Serum samples from 49 patients diagnosed with pSS, 33 patients with rheumatoid arthritis (RA), and 30 healthy controls (HCs) were collected for measurements of CXCL9, CXCL10, CXCL11, and CXCR3. Additionally, CXCL levels in the MSG tissues were measured in 41 patients who underwent MSG biopsy. Correlations between CXCL and CXCL/CXCR levels in serum/MSG tissues and clinical factors/salivary scintigraphy parameters were analyzed. Serum CXCL11 and CXCR3 showed statistically significant differences among patients with pSS and RA and HCs (serum CXCL11, pSS:RA:HC = 235.6 ± 500.1 pg/mL:90.0 ± 200.3 pg/mL:45.9 ± 53.6 pg/mL; *p* = 0.041, serum CXCR3, pSS:RA:HC = 3.27 ± 1.32 ng/mL:3.29 ± 1.17 ng/mL:2.00 ± 1.12 ng/mL; *p* < 0.001). Serum CXCL10 showed a statistically significant difference between pSS (64.5 ± 54.2 pg/mL) and HCs (18.6 ± 18.1 pg/mL, *p* < 0.001), while serum CXCL9 did not exhibit a significant difference among the groups. Correlation analysis of clinical factors revealed that serum CXCL10 and CXCL11 levels positively correlated with erythrocyte sedimentation rate (*r* = 0.524, *p* < 0.001 and *r* = 0.707, *p* < 0.001, respectively), total protein (*r* = 0.375, *p* = 0.008 and *r* = 0.535, *p* < 0.001, respectively), globulin (*r* = 0.539, *p* < 0.001 and *r* = 0.639, *p* < 0.001, respectively), and European Alliance of Associations for Rheumatology SS Disease Activity Index (*r* = 0.305, p = 0.033 and *r* = 0.321, *p* = 0.025). Additionally, serum CXCL10 negatively correlated with the Schirmer test score (*r* = − 0.354, *p* = 0.05), while serum CXCL11 positively correlated with the biopsy focus score (*r* = 0.612, *p* = 0.02). In the MSG tissue, the percentage of infiltrating CXCL9-positive cells was highest (75.5%), followed by CXCL10 (29.1%) and CXCL11 (27.9%). In the correlation analysis, CXCL11-expressing cells were inversely related to the mean washout percentage on salivary gland scintigraphy (*r* =  − 0.448, *p* = 0.007). Our study highlights distinct serum and tissue chemokine patterns in pSS, emphasizing CXCL9’s potential for early diagnosis. This suggests that CXCL10 and CXCL11 are indicators of disease progression, warranting further investigation into their roles in autoimmune disorders beyond pSS.

## Background

Sjogren’s syndrome (SS) is an autoimmune disorder that manifests as dryness of the eyes and mouth accompanied by lymphocytic infiltrates in the lacrimal and salivary glands. This chronic condition, categorized as primary (pSS) or secondary SS, is often linked to other rheumatic diseases and presents with extraglandular manifestations across various systems [1]. The diagnosis of SS relies on a combination of clinical, serological, and functional tests and histopathological biomarkers based on the 2016 American College of Rheumatology/European Alliance of Associations for Rheumatology (ACR/EULAR) classification criteria [2]. Histological examination is crucial in seronegative cases, where approximately 20–30% of patients may not be positive for specific autoantibodies, such as antinuclear antibody (ANA), anti-Ro/SSA, and anti-La/SSB [3]. Despite being considered minimally invasive, a salivary gland biopsy may lead to complications, including pain and sensory impairment [4]. For these reasons, some patients may refuse this procedure, prompting an ongoing debate on whether mandatory tissue testing should be implemented for disease diagnosis [5]. Due to all these circumstances, the diagnosis of pSS remains complex and challenging.

Some individuals may not exhibit apparent symptoms of dryness, a key indicator of SS, whereas some healthcare professionals may not immediately recognize the association between dryness symptoms and the disease, leading to a delayed diagnosis of pSS. In recent years, there has been growing interest in identifying biomarkers that can aid in the early diagnosis of pSS and provide insights into its pathogenesis and disease activity. Among these potential candidates, the C-X-C motif chemokine ligand (CXCL), a key player in inflammatory and immune responses, has emerged as a promising candidate [6]. The chemokines CXCL9, CXCL10, and CXCL11, known for their function in coordinating the directional migration of CD4 + TH1, CD8 + T, natural killer (NK), and NKT cells through interaction with their mutual receptor, CXC chemokine receptor 3 (CXCR3), are implicated in various pathophysiological processes of autoinflammation and autoimmune diseases [7, 8].

These molecules have gained attention as potential therapeutic targets for various diseases [9, 10]. Therefore, this study aimed to explore and elucidate the roles of CXCL9, CXCL10, CXCL11, and CXCR3 in the pathophysiology of pSS, highlighting on their potential as diagnostic and prognostic biomarkers. We assessed the expression levels of various molecules in the serum and salivary glands of patients with pSS and investigated their associations with clinical symptoms.

## Methods

### Study population

This single-center study was conducted in the Department of Rheumatology at the Ajou University. The study protocol was approved by the Ajou University Hospital Institutional Review Board (AJIRB-SMP-2022-265). All participants understood the study and provided consent to participate in this study, which was conducted in accordance with the principles of the Declaration of Helsinki. We included patients aged ≥ 18 years who were diagnosed with pSS between July 2022 and June 2023. The diagnosis of pSS followed the 2016 ACR/EULAR classification criteria [2]. Exclusion criteria comprised patients with other autoimmune diseases (rheumatoid arthritis [RA], systemic lupus erythematosus, systemic sclerosis, or immunoglobulin G4-related disease) and those prescribed muscarinic agonists (pilocarpine and cevimeline). We obtained serum samples from 33 patients with RA and 30 healthy controls (HC).

Demographic, clinical, and laboratory data related to pSS were extracted from medical records. Disease activity was assessed using the EULAR Sjogren’s syndrome disease activity index (ESSDAI) at the time of sample collection. The ESSDAI, ranging between 0–123, encompasses 12 domains (cutaneous, respiratory, renal, articular, muscular, peripheral nervous system, central nervous system, hematological, glandular, constitutional, lymphadenopathic, and biological), categorized as low (ESSDAI < 5), moderate (5 ≤ ESSDAI ≤ 13), and high disease (ESSDAI ≥ 14) activities [11].

Assessment of dry eye symptoms involved the utilization of the Schirmer test, with a result of < 5 mm absorption on at least one Schirmer strip after 5 min considered abnormal. Dry mouth was evaluated by collecting unstimulated whole saliva (UWS) and stimulated whole saliva (SWS), with salivary flow rates recorded in mL/min. Unstimulated whole saliva was collected for 10 min using the spitting method, while SWS was obtained by chewing paraffin gum, which involved discarding the saliva produced in the initial 2 min and using the subsequent 5 min collection for analysis. Salivary gland scintigraphy was performed to quantify the status of salivary gland secretion and excretion. Patients were administered ^99m^Tc-pertechnetate, followed by measurement of radiotracer distribution using a dual-head gamma camera (GE Healthcare, USA). Circular regions of interest were drawn on each parotid and submandibular gland after glandular stimulation, and uptake and excretion ratios were assessed semiquantitatively.

### Evaluation of serum CXCL9, CXCL10, CXCL11, and CXCR3 levels

Serum levels of CXCL9, CXCL10, CXCL11, and CXCR3 were measured using commercially available ELISA kits (R&D Systems, Minneapolis, MN, USA). *All serum samples were measured without dilution. The assay ranges for obtaining reliable results with each kit were as follows: for CXCL9, the range was 62.5–4000 pg/mL; for CXCL10, the range was 31.2–2000 pg/mL; for CXCL11, the range was 7.8–500 pg/mL; and for CXCR3, the range was 0.16–10 ng/mL*. The kit provided all necessary materials, and the test was conducted in accordance with the manufacturer’s instructions.

### Histopathological analysis and staining evaluation

Salivary gland biopsies were obtained from 41 patients with pSS, and hematoxylin and eosin-stained sections were examined. *All slides were assessed by three expert pathologist (JHH, JEK, and HY), who were blinded to clinical information*. Extent of lymphocytic infiltration was quantified using the focus score, with > 50 periductal lymphocytes in 4 mm^2^ of salivary gland tissue defining a focus [12]. Interstitial fibrosis is characterized by collagenous fibrosis surrounding the ducts or formation of tracts that separate lobules and encapsulated acini [13]. Slides were scored based on the severity of interstitial fibrosis as follows: 0 = no or minimal fibrosis, 1 = mild fibrosis without acinar replacement, 2 = moderate fibrosis with acinar replacement, and 3 = severe fibrosis with acinar replacement and marked gland disruption. We also determined the presence of germinal center-like structures, characterized by densely aggregated lymphoid cells containing distinguishable dark and light zones within the minor salivary glands.

Immunohistochemical staining was conducted using BenchMark XT (Ventana Medical Systems, AZ, USA), following standard protocols. Tissue sections, cut to a thickness of 4 μm, were deparaffinized in xylene and dehydrated in graded ethanol. Antigen retrieval was performed using Cell Conditioning Buffer 1 for 40 min. Primary antibodies used in this study included CXCL9 (1:50), CXCL10 (1:50), and CXCL11 (1:50) obtained from R&D Systems. *Scores were determined by dividing the number of positive inflammatory cells by the total number of inflammatory cells and expressed as percentages of CXCL9, CXCL10, and CXCL11. Each chemokine was stained and read on a single slide per chemokine*.

### Statistical analysis

Continuous variables were analyzed using either the Student’s t-test or Mann–Whitney U test, while categorical variables were assessed using the Chi-square or Fisher’s exact test. Mean values accompanied by standard deviations characterized the presentation of continuous variables, and statistical significance was determined at *p* < 0.05. *We performed one-way ANOVA to compare the serum levels of CXCL9, CXCL10, CXCL11, and CXCR3 among the three groups. Post hoc pairwise comparisons were conducted using Tukey's HSD test*. The Spearman’s correlation analysis was performed to explore the associations between clinical parameters, disease activity, and salivary gland histological data. The strength of these correlations was categorized based on the Spearman’s correlation coefficient (*r*): very weak (0.0–0.2), weak (0.2–0.4), moderate (0.4–0.6), strong (0.6–0.8), or very strong (0.8–1.0). All statistical analyses were performed using SPSS software (version 25.0; IBM Corporation, Armonk, NY, USA).

## Results

### Patient characteristics

The study included 49 patients with pSS, 30 patients with RA, and 33 HCs; their characteristics are shown in Table 1. Mean ages of the pSS, RA, and HC groups were 47.6 ± 11.3, 48.5 ± 10.1, and 43.3 ± 7.3 years, respectively, with no significant differences observed among the three groups. The proportions of females were 95.9%, 90%, and 90.9% in the pSS, RA, and HC groups, respectively, with no significant differences. Upon reviewing information of the 49 patients with pSS, mean duration of symptoms was 4.8 ± 5.0 years, with all of them (100%) experiencing ocular dryness and 45 (91.8%) experiencing oral dryness symptoms. Fatigue was the predominant extraglandular symptom reported in 79.6% of the cases, followed by arthralgia (51.0%), purpura (24.5%), and arthritis (14.3%). Disease activity of pSS was assessed using the ESSDAI score, with a mean score of 3.67 ± 2.15. Thirteen (26.5%) patients had moderate to high disease activity, defined as ESSDAI ≥ 5. Mean results of the Schirmer test for dry eyes were 6.19 ± 6.80 mm on the left side and 5.45 ± 5.20 mm on the right side. Mean salivary flow rates of UWS and SWS were 0.08 ± 0.13 and 0.49 ± 0.51 mL/min, respectively. Laboratory findings revealed ANA positivity in 46 (93.9%) patients and rheumatoid factor (RF) positivity in 30 (61.2%). Anti-SSA/Ro was positive in 44 (89.8%) patients, and 11 (22.4%) tested positive for anti-SSB/La.

**Table 1:** Baseline characteristics

Variable	pSS patients (*n* = 49)	RA (*n* = 30)	HC (*n* = 33)	*p*-value
Age, mean, years	47.6 ± 11.3	48.5 ± 10.1	43.3 ± 7.3	0.079
Female, no. (%)	47 (95.9)	27 (90)	30 (90.9)	0.535
Symptom duration, mean, years	4.8 ± 5.0			
BMI, mean, kg/m^2^	22.6 ± 4.6			
Glandular manifestations				
Ocular symptoms, no. (%)	49 (100)			
Oral symptoms, no. (%)	45 (91.8)			
Extraglandular manifestations				
Photosensitivity, no. (%)	3 (6.1)			
Arthralgia, no. (%)	25 (51.0)			
Arthritis, no. (%)	7 (14.3)			
Raynaud’s phenomenon, no. (%)	4 (8.2)			
Purpura, no. (%)	12 (24.5)			
Fatigue, no. (%)	39 (79.6)			
ESSDAI	3.67 ± 2.15			
ESSDAI ≥ 5, no. (%)	13 (26.5)			
DAS28		2.66 ± 1.17		
Schirmer test, left, mm	6.19 ± 6.80			
Schirmer test, right, mm	5.45 ± 5.20			
Salivary flow, UWS, mL/min	0.076 ± 0.126			
Salivary flow, SWS, mL/min	0.491 ± 0.508			
Laboratory data				
Leukocyte, /μL (normal range 3,400 ‒ 10,600)	4969.2 ± 1929.5	5541 ± 2112.8		0.221
Hemoglobin, g/dL (normal range 12.5 ‒ 17.5)	12.7 ± 1.16	12.9 ± 1.39		0.656
Platelet, 10^3^/μL (normal range 134 ‒ 387)	238.3 ± 56.2	236.2 ± 67.4		0.881
ESR, mm/hr (normal range 0 ‒ 25)	27.3 ± 22.8	16.7 ± 11.4		**0.008**
CRP, mg/dL (normal range 0 ‒ 0.5)	0.34 ± 0.88	0.30 ± 0.58		0.863
Total protein, g/dL (normal range 6.6 ‒ 8.7)	7.8 ± 0.77	7.17 ± 0.43		** < 0.001**
Albumin, g/dL (normal range 3.5 ‒ 5.2)	4.5 ± 0.28	4.46 ± 0.21		0.525
RF positivity, no. (%)	30 (61.2)	28 (93.3)		**0.002**
ANA positivity, no. (%)	46 (93.9)			
Anti-SSA/Ro positivity, no. (%)	44 (89.8)			
Anti-SSB/La positivity, no. (%)	11 (22.4)			

Mean ± SD. pSS, primary Sjogren’s syndrome; RA, rheumatoid arthritis; HC, healthy control; BMI, body mass index; RTA, renal tubular acidosis; ESSDAI, European League Against Rheumatism Sjogren’s Syndrome disease activity index; DAS28, disease activity score of 28 joints; UWS, unstimulated whole saliva; SWS, stimulated whole saliva; ESR, erythrocyte sedimentation rate; CRP, c-reactive protein; RF, rheumatoid factor; ANA, antinuclear antibody

Bold values indicate significant *p*-value

### Minor salivary gland biopsy and salivary gland scintigraphy

Table 2 presents data from 41 patients who underwent minor salivary gland biopsy and salivary gland scintigraphy. In the minor salivary gland tissue examination, a mean of 73,465.7 ± 145,097.8 cell detections were recorded. The mean focus score per 4 mm^2^ was 1.32 ± 1.67, with 27 (65.9%) patients having a focus score of ≥ 1. Additionally, only two (4.9%) patients showed the formation of germinal centers. Salivary gland scintigraphy evaluated the peak uptake ratio, mean uptake ratio, and percentage washout of both the parotid and submandibular glands. The mean values for peak uptake and mean uptake of the bilateral parotid and submandibular glands were 5.14 ± 1.63 and 3.35 ± 1.08, respectively. The mean percentage washout of the bilateral parotid and submandibular glands was 30.5 ± 17.7.

**Table 2:** Histopathological and salivary gland scintigraphy parameters in patients with pSS

Variable	pSS patients (*n* = 41)
Histopathological parameters	
Number of cell detections	73,465.7 ± 145,097.8
Total focus score	2.71 ± 6.62
Focus score per 4mm^2^	1.32 ± 1.67
Focus score/4mm^2^ ≥ 1, no. (%)	27 (65.9)
Germinal center positive, no. (%)	2 (4.9)
Salivary gland scintigraphy parameters	
Peak uptake ratio	
Right parotid gland	5.65 ± 2.38
Left parotid gland	5.28 ± 2.11
Right submandibular gland	4.86 ± 1.66
Left submandibular gland	4.77 ± 1.57
Mean	5.14 ± 1.63
Mean uptake ratio	
Right parotid gland	3.62 ± 1.48
Left parotid gland	3.32 ± 1.31
Right submandibular gland	3.27 ± 1.14
Left submandibular gland	3.17 ± 1.07
Mean	3.35 ± 1.08
% wash out	
Right parotid gland	35.2 ± 17.5
Left parotid gland	35.2 ± 17.6
Right submandibular gland	26.0 ± 14.7
Left submandibular gland	25.0 ± 15.7
Mean	30.5 ± 17.7

Mean ± SD; pSS, primary Sjogren’s syndrome; LCA, leukocyte common antigen

### Comparison of serum CXCL9, CXCL10, CXCL11, and CXCR3 levels in pSS, RA, and HCs

**Fig. 1:**
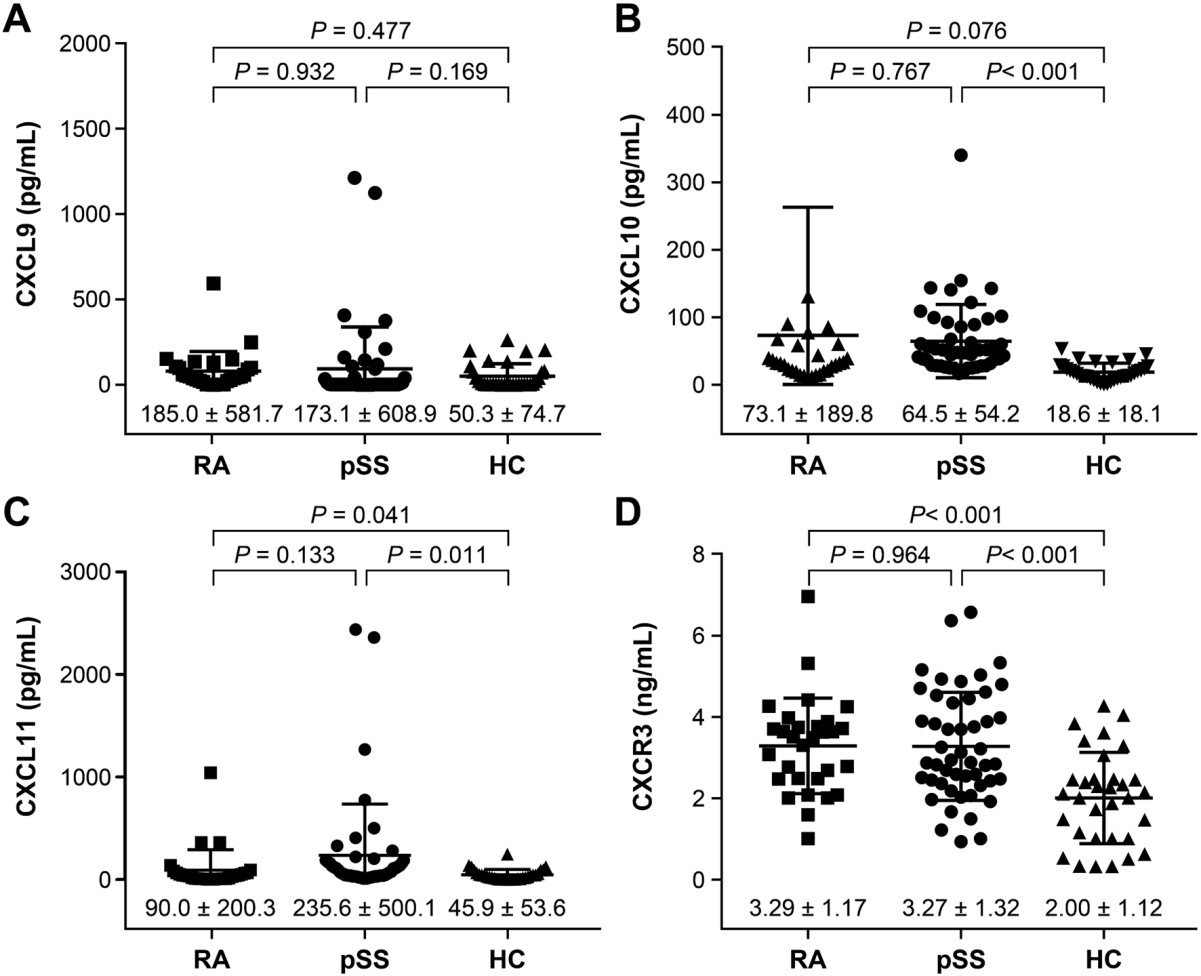
Serum levels of CXCL9 (**a**), CXCL10 (**b**), CXCL11 (**c**), and CXCR3 (**d**) in primary Sjögren’s syndrome (pSS), rheumatoid arthritis (RA), and healthy controls (HCs). *Data are expressed as means ± standard deviations and were analyzed using one-way ANOVA to compare the serum levels of CXCL9, CXCL10, CXCL11, and CXCR3 among the three groups. Post hoc pairwise comparisons were conducted using Tukey's HSD test*

Figure 1 illustrates the comparison of serum CXCL9, CXCL10, CXCL11, and CXCR3 levels between patients with pSS, RA, and HCs. Serum CXCL9 levels were 173.1 ± 608.9 pg/mL for pSS, 185.0 ± 581.7 pg/mL for RA, and 50.3 ± 74.7 pg/mL for HCs, with no significant differences observed between the groups. Serum CXCL10 levels were 64.5 ± 54.2 pg/mL for pSS, 73.1 ± 189.8 pg/mL for RA, and 18.6 ± 18.1 pg/mL for HCs, with a significant difference between pSS and HCs (*p* < 0.001). Serum CXCL11 levels were 235.6 ± 500.1 pg/mL for pSS, 90.0 ± 200.3 pg/mL for RA, and 45.9 ± 53.6 pg/mL for HCs, with significant differences between the three groups (*p* < 0.001). Pairwise comparisons showed a significant difference between pSS and HCs (*p* = 0.011). Serum CXCR3 levels were 3.27 ± 1.32 ng/mL for pSS, 3.29 ± 1.17 ng/mL for RA, and 2.00 ± 1.12 ng/mL for HCs, with significant differences between the three groups (*p* < 0.001), as well as between pSS and HCs (*p* < 0.001).

### Correlations between serum CXCL9, CXCL10, CXCL11, and CXCR3 levels with clinical factors of pSS

We conducted an analysis to determine the correlation between clinical factors and serum levels of CXCL9, CXCL10, CXCL11, and CXCR3 (Table 3). Serum CXCL9 levels showed significant positive correlation with the ESSDAI scores (*r* = 0.351, *p* = 0.013). Serum CXCL10 levels showed significant positive correlations with erythrocyte sedimentation rate (ESR) (*r* = 0.524, *p* < 0.001), total protein (*r* = 0.375, *p* = 0.008), globulin (*r* = 0.539, *p* < 0.001), and ESSDAI (*r* = 0.305, *p* = 0.033), and negative correlation with the Schirmer test (*r* = ** − **0.354, *p* = 0.05). Furthermore, CXCL11 showed significant positive correlations with focus score (*r* = 0.612, *p* = 0.020), ESR (*r* = 0.707, *p* < 0.001), total protein (*r* = 0.535, *p* < 0.001), globulin (*r* = 0.639, *p* < 0.001), RF (*r* = 0.463, *p* = 0.005), and ESSDAI (*r* = 0.321, *p* = 0.025). Serum CXCR3 levels showed significant positive correlation with C-reactive protein (CRP) levels (*r* = 0.394, *p* = 0.005).

**Table 3:** Correlations between serum CXCL with clinical factors of pSS

Clinical factors	Correlation coefficient, *r* (*p*-value)			
	CXCL9	CXCL10	CXCL11	CXCR3
Focus score	− 0.246 (0.397)	− 0.065 (0.827)	**0.612 (0.020)**	0.441 (0.115)
Mean peak uptake	0.261 (0.367)	− 0.160 (0.584)	− 0.242 (0.404)	− 0.356 (0.211)
Mean mean uptake	0.153 (0.601)	− 0.240 (0.409)	− 0.332 (0.246)	− 0.356 (0.211)
Mean % washout	0.291 (0.313)	− 0.332 (0.246)	− 0.418 (0.137)	− 0.169 (0.563)
Leukocyte	− 0.182 (0.210)	− 0.189 (0.193)	− 0.169 (0.246)	− 0.011 (0.939)
Hemoglobin	− 0.005 (0.972)	− 0.052 (0.722)	− 0.079 (0.592)	− 0.013 (0.927)
ESR	0.249 (0.085)	**0.524 (< 0.001)**	**0.707 (< 0.001)**	− 0.006 (0.967)
CRP	0.108 (0.458)	0.089 (0.545)	0.200 (0.169)	**0.394 (0.005)**
Total protein	− 0.065 (0.659)	**0.375 (0.008)**	**0.535 (< 0.001)**	0.102 (0.484)
Globulin	0.036 (0.806)	**0.539 (< 0.001)**	**0.639 (< 0.001)**	0.148 (0.311)
Complement 3	− 0.203 (0.180)	− 0.162 (0.279)	− 0.045 (0.770)	− 0.100 (0.514)
Complement 4	− 0.078 (0.609)	− 0.009 (0.952)	0.121 (0.428)	− 0.046 (0.762)
RF	0.085 (0.626)	0.219 (0.207)	**0.463 (0.005)**	0.064 (0.716)
ESSDAI	**0.351 (0.013)**	**0.305 (0.033)**	**0.321 (0.025)**	− 0.217 (0.134)
Schirmer test, mean	− 0.101 (0.587)	− **0.354 (0.050)**	− 0.237 (0.200)	− 0.189 (0.308)
SWS flow rates	0.018 (0.926)	− 0.173 (0.379)	− 0.318 (0.099)	− 0.127 (0.519)
UWS flow rates	− 0.069 (0.733)	− 0.249 (0.210)	− 0.171 (0.393)	− 0.240 (0.229)

CXCL, C-X-C motif chemokine ligands; CXCR, C-X-C motif chemokine receptor; pSS, primary Sjogren’s syndrome; ESR, erythrocyte sedimentation rate; CRP, c-reactive protein; RF, rheumatoid factor; ESSDAI, European League Against Rheumatism Sjogren’s Syndrome disease activity index; SWS, stimulated whole saliva; UWS, unstimulated whole saliva

Bold values indicate significant *p*-value

### Immunohistochemical data

**Fig. 2:**
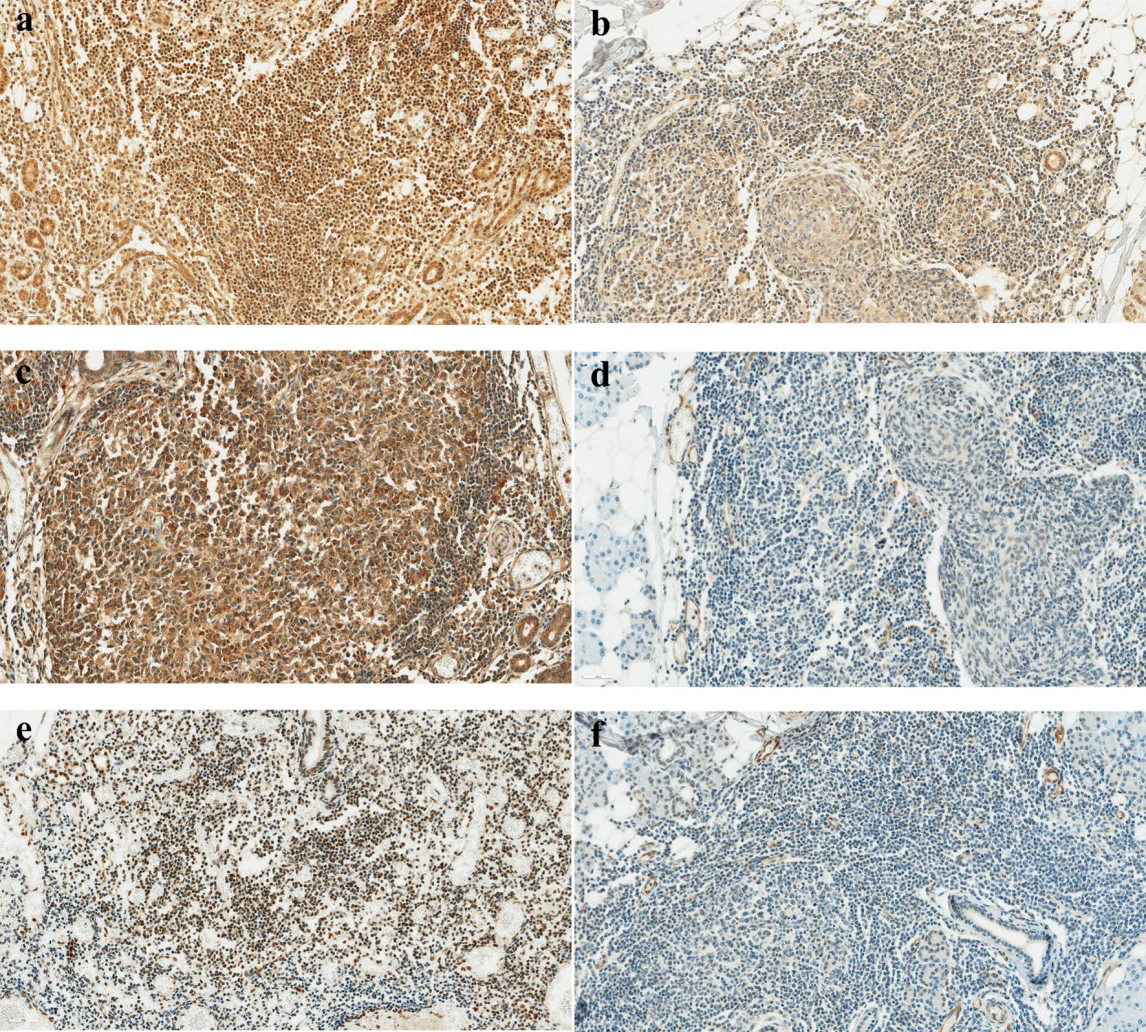
Immunohistochemical staining of high and low CXCL expression in minor salivary gland tissue of patients with Sjögren’s Syndrome (× 400). **a** High CXCL9 expression, **b** Low CXCL9 expression, **c** High CXCL10 expression, **d** Low CXCL10 expression, **e** High CXCL11 expression, and **f** Low CXCL11 expression

We immunohistochemically evaluated the expression of CXCL9, CXCL10, and CXCL11 in the minor salivary gland tissues of patients with pSS (Fig. 2). The percentage of cells expressing CXCL9 was 75.5 ± 18.7%, while that of CXCL10 was 29.1 ± 27.9%. Additionally, the percentage of cells expressing CXCL11 was 27.9 ± 20.8%. The results of the investigation of the relationship between the expression levels of each chemokine in the tissues and clinical manifestations are presented in Table 4. We observed a significant negative correlation between the percentage of cells expressing CXCL9 and leukocyte (*r* =  − 0.355, *p* = 0.025), CRP (*r* = 0.331, *p* = 0.042), as well as complement 3 (*r* = ** − **0.487, *p* = 0.006). Furthermore, the percentage of cells expressing CXCL11 showed a significant negative correlation with the mean percentage washout of the bilateral parotid and submandibular glands (*r* = ** − **0.448, *p* = 0.007).

**Table 4:** The correlation between chemokine expression in minor salivary gland tissue and clinical factors of pSS

Clinical factors	Correlation coefficient, *r* (*p*-value)		
	CXCL9	CXCL10	CXCL11
Focus score	0.043 (0.791)	0.010 (0.953)	0.045 (0.780)
Mean peak uptake	− 0.352 (0.038)	0.072 (0.681)	− 0.280 (0.103)
Mean mean uptake	− 0.285 (0.097)	0.087 (0.621)	− 0.238 (0.169)
Mean % washout	− 0.294 (0.086)	0.026 (0.881)	− **0.448 (0.007)**
Leukocyte	− **0.355 (0.025)**	0.002 (0.992)	− 0.262 (0.102)
Hemoglobin	0.153 (0.346)	0.202 (0.212)	− 0.025 (0.880)
ESR	− 0.247 (0.135)	− 0.243 (0.142)	0.087 (0.602)
CRP	**0.331 (0.042)**	0.073 (0.664)	− 0.234 (0.158)
Total protein	− 0.017 (0.919)	− 0.030 (0.857)	0.213 (0.198)
Globulin	− 0.045 (0.788)	− 0.127 (0.446)	0.254 (0.123)
Complement 3	− **0.487 (0.006)**	− 0.307 (0.099)	− 0.128 (0.501)
Complement 4	− 0.205 (0.278)	− 0.164 (0.388)	0.030 (0.874)
RF	− 0.120 (0.468)	0.066 (0.690)	0.009 (0.956)
ESSDAI	0.172 (0.284)	− 0.093 (0.562)	0.094 (0.561)
Schirmer test, mean	− 0.206 (0.428)	− 0.080 (0.760)	0.139 (0.594)
SWS flow rates	− 0.046 (0.819)	0.084 (0.678)	0.111 (0.581)
UWS flow rates	− 0.156 (0.438)	0.116 (0.566)	0.213 (0.287)

CXCL, C-X-C motif chemokine ligands; pSS, primary Sjogren’s syndrome; ESR, erythrocyte sedimentation rate; CRP, c-reactive protein; RF, rheumatoid factor; ESSDAI, European League Against Rheumatism Sjogren’s Syndrome disease activity index; SWS, stimulated whole saliva; UWS, unstimulated whole saliva

Bold values indicate significant *p*-value

## Discussion

The significant involvement of CXCL chemokines and their CXCR in the pathophysiology of autoimmune diseases, including pSS, is underscored by the facilitation of inflammatory cytokine production and promotion of inflammatory responses [14]. Among these, CXCL10 notably relies on IFN-γ for secretion, playing a pivotal role in promoting Th1 cell activation and amplifying inflammation through CXCR3-mediated feedback loops, thereby significantly influencing immune regulation in autoimmune diseases [15–17]. Extensive studies has been conducted on the role of CXCL10 in the onset and progression of pSS, with studies reporting enhanced CXCL10 expression in salivary gland ducts and acinar cells stimulated by IFN-γ [18–20]. Alongside CXCL10, the CXCL9 and CXCL11 axes play crucial roles in regulating immune cell trafficking, differentiation, and activation [9]. Although some studies have highlighted the regulatory roles of each component in patients with pSS, few studies have addressed these aspects within the same patient cohort [21, 22]. Therefore, in this study, we comprehensively examined the expression levels of CXCL9, CXCL10, CXCL11, and CXCR3 in the sera of patients with pSS and further explored their expression levels in minor salivary glands.

Comparative analysis of serum chemokine levels revealed distinct patterns in patients with pSS compared to those in patients with RA and HCs. Serum CXCR3 and CXCL11 levels were notably higher in patients with pSS than those in patients with RA and HCs, showing the most significant differences among the three groups. Additionally, serum CXCL10 levels were significantly elevated in patients with pSS compared with those in HCs. These findings demonstrate a trend similar to that reported in other studies, indicating a potential role for the CXCL10 and 11/CXCR3 axes in the pathogenesis of pSS [23, 24]. When comparing only the pSS and RA groups, no significant differences were observed; similarly, these chemokines have been reported to increase not only in patients with pSS but also in the serum and synovial fluid of those with RA [25]. The overlap of specific clinical and serological signs between pSS and RA implies shared pathophysiological mechanisms in the physiology of both diseases, making it challenging to differentiate between the two conditions based solely on serum chemokine levels [26].

Contrary to other studies, serum CXCL9 levels in pSS did not show significant differences compared with RA and HC in this study [27]. In previous studies using patients with pSS or SS-like mouse models, CXCL9 expression increased significantly in most cases [27, 28]. In studies similar to ours, insignificant results were obtained, possibly because of accompanying clinical features or serological characteristics [29]. Factors, such as lower disease activity among the patients enrolled in our study and prolonged use of immunosuppressive agents, may have influenced the chemokine levels. Furthermore, although CXCL9, CXCL10, and CXCR3 levels were comparable between patients with pSS and RA in our study, the notably higher levels of CXCL11 in patients with pSS suggest that CXCL11 may contribute more dramatically to CXCR3-binding affinity or IFN-inducible CXCR3 ligand activity than CXCL9 and CXCL10 [30, 31].

Furthermore, CXCL11 showed the strongest association with the clinical manifestations of pSS, exhibiting a strong correlation with the lymphocytic focus score in minor salivary gland biopsies. Since previous studies have found no relationship between the levels of serum chemokines and lymphocytic focus scores in tissues, the use of serum CXCL11 should be further emphasized in patients for whom tissue examination is not feasible. In biomarker discovery studies targeting patients with pSS, associations with clinical symptoms or disease activity have been reported in most cases [32], and the molecules included in our study demonstrated similar findings. In addition to CXCL11, both CXCL10 and CXCR3 have been significantly linked to an increase in inflammatory and immune activity, as indicated by elevated ESR/CRP levels, total protein, globulin, and ESSDAI. This suggests their potential utility as disease biomarkers for clinical monitoring and as indicators of pSS onset.

However, numerous reports have indicated elevated serum CXCL levels not only in pSS but also in other autoimmune diseases, diminishing their use as disease-specific biomarkers [33–35]. Consequently, recent studies focus on measuring the degree of infiltration within organs that are directly involved in the disease process. We also investigated the role of CXCL 9/10/11 in the salivary glands by measuring the proportion of cells expressing each chemokine. Unlike serum CXCL levels, the percentage of cells positive for CXCL9 was highest in the salivary gland, with CXCL10 and CXCL11 showing sufficiently high positivity rates of around 25–30%. Although there were no studies measuring CXCL levels in patients with pSS using the same methodology as our current study, comparing with studies from other conditions, we found a significantly higher proportion of inflammatory cells expressing CXCL9 [36, 37]. Serum CXCL9 levels were not significantly different between HCs and patients with pSS; however, the marked increase in CXCL9 expression in the salivary glands may indicate a discrepancy in the timing of CXCL expression relative to disease progression. Thus, CXCL9 appears to be expressed both before and during the disease peak, whereas CXCL10 expression reportedly increases as the disease progresses, implying a sequential expression pattern for CXCL9 and CXCL10 [38]. In our study, salivary gland biopsies in patients with pSS were conducted at very early stages for diagnostic purposes, likely resulting in the abundant expression of CXCL9. Conversely, serum level measurements were performed during the follow-up period after disease progression, leading to a significant difference in CXCL10 compared to that in HCs, rather than CXCL9. The disparity in timing between tissue examination and serum sample collection likely contributed to these findings.

The higher the expression levels of each chemokine in the tissue, the better they reflected the clinical symptoms. In this study, the tissue expression level of CXCL9 was found to correlate with the inflammation and hematologic abnormalities observed in pSS, which is consistent with previous reports, indicating that CXCL9 is more significantly expressed in pSS patients with extraglandular manifestations [27]. Although CXCL10 and CXCL11 showed a relatively lower capability to reflect clinical symptoms than CXCL9 in minor salivary gland tissue, significant findings emerged, indicating that CXCL11 expression increases notably with severe salivary gland dysfunction, as confirmed by salivary gland scintigraphy. Our findings underscore the significance of CXCL9, 10, and 11 as key components of inflammatory response in the salivary glands of patients with pSS and suggest their potential roles as biomarkers for diagnosis and disease activity in symptomatic tissues. *Additionally, through tissue immunohistochemistry, our study supports the significant involvement of T cells and monocytes in the pathogenesis of pSS, consistent with prior research *[39]. Several studies, including ours, have reported increased levels of CXCL9, 10, and 11 in salivary gland lesions [21, 40, 41], with similar findings observed in other tissues where dryness symptoms can manifest in patients with pSS, including the cervix, vagina, and tear glands [42, 43]. Additionally, there have been reports of increased expression levels of CXCL9/10/11 in the conjunctival epithelium of pSS patients with xerophthalmia [44].

This study has strengths. To our knowledge, this study is the first to measure various chemokines in the serum and minor salivary gland tissues of Korean patients with pSS. However, this study has several limitations. *First, there was a lack of matching between the group that measured serum chemokines and the group that measured chemokines in minor salivary gland tissues, as well as a difference in timing between serum sample collection and tissue biopsy. Due to the retrospective nature of this study, the timing of serum samples was not consistent. Samples from patients who were not newly diagnosed were also included, potentially influencing chemokine levels.* Prospective studies measuring the levels in both the serum and minor salivary gland tissues at the time of diagnosis are needed to confirm the use of each chemokine as a diagnostic and disease activity biomarker. Second, we could not confirm CXCR3 expression in minor salivary gland tissues. Due to insufficient tissue remaining after the diagnostic biopsy, CXCR3 staining could not be performed. *Additionally, it was not feasible to request additional biopsies from patients solely for CXCR3 staining, as this invasive procedure can cause pain and nerve damage, and using tissues not obtained at the same time would not provide accurate analyses. Third, the patients may have received immunosuppressive therapy, and serum chemokine sampling during the course of treatment may have been significantly influenced. We additionally stratified patients with pSS based on the use of immunosuppressive agents and measured serum levels of CXCL9, CXCL10, CXCL11, and CXCR3 (data are not shown). Although levels after treatment did not show statistical significance, this suggests that the use of immunosuppressants had minimal impact on our findings. Longer therapy duration may have greater effects, warranting prospective and follow-up studies to explore chemokine level changes and their relationship with disease activity. Finally, the immunohistochemistry methods we utilized for minor salivary glands did not enable us to fully elucidate the precise roles of lymphocyte subsets*. However, by elucidating their significant roles in the serum and minor salivary glands of patients with pSS, this study may serve as a foundation for future pSS biomarker study on pSS.

## Conclusions

Our study highlights the intricate roles of chemokines in pSS and provides valuable insights into their potential as diagnostic and disease activity biomarkers. We observed distinct patterns in serum chemokine levels, with CXCR3 and CXCL11 showing significant differences between patients with pSS, RA, and HCs. Additionally, CXCL9 exhibited the highest expression in salivary gland tissues. An increase in serum and tissue chemokine expression was significantly associated with disease activity in pSS. Although consistent results were not obtained between serum and tissue samples, this discrepancy is believed to stem from differences in sampling timing and the varied timing of chemokine upregulation across tissues. Overall, CXCL9 is presumed to play a more significant role in aiding early diagnosis, whether in the serum or tissues, whereas CXCL10 and CXCL11 are considered indicators that better reflect disease progression or activity during follow-up. Although challenges, such as overlapping clinical features with other autoimmune diseases and treatment influences exist, our findings can pave the way for future studies aimed at validating these chemokines as reliable indicators of pSS onset and progression.

## Data Availability

All available data are reported in the manuscript and supplementary file.

## Abbreviations

SS: Sjogren’s syndrome
pSS: Primary Sjogren’s syndrome
ACR: American College of Rheumatology
EULAR: European Alliance of Associations for Rheumatology
ANA: Antinuclear antibody
HC: Healthy control
CXCL: C-X-C motif chemokine ligand
CXCR: CXC receptor
MSGs: Minor salivary glands
RA: Rheumatoid arthritis
NK: Natural killer
ESSDAI: EULAR Sjogren’s syndrome disease activity index
UWS: Unstimulated whole saliva
SWS: Stimulated whole saliva
RF: Rheumatoid factor
ESR: Erythrocyte sedimentation rate
CRP: C-reactive protein
